# A Possible Role of the Full-Length Nascent Protein in Post-Translational Ribosome Recycling

**DOI:** 10.1371/journal.pone.0170333

**Published:** 2017-01-18

**Authors:** Debasis Das, Dibyendu Samanta, Arpita Bhattacharya, Arunima Basu, Anindita Das, Jaydip Ghosh, Abhijit Chakrabarti, Chanchal Das Gupta

**Affiliations:** 1 Department of Biophysics, Molecular Biology and Bioinformatics, University College of Science, University of Calcutta, Kolkata, India; 2 Department of Microbiology, Raidighi College, Raidighi, 24 Parganas (S), West Bengal, India; 3 Department of Microbiology, St. Xavier’s College, Kolkata, India; 4 Crystallography & Molecular Biology Division, Saha Institute of Nuclear Physics, HBNI, Kolkata, India; 5 Department of Life Sciences and Biotechnology, Jadavpur University, Kolkata, India; Universidad Nacional Autonoma de Mexico, MEXICO

## Abstract

Each cycle of translation initiation in bacterial cell requires free 50S and 30S ribosomal subunits originating from the post-translational dissociation of 70S ribosome from the previous cycle. Literature shows stable dissociation of 70S from model post-termination complexes by the concerted action of Ribosome Recycling Factor (RRF) and Elongation Factor G (EF-G) that interact with the rRNA bridge B2a/B2b joining 50S to 30S. In such experimental models, the role of full-length nascent protein was never considered seriously. We observed relatively slow release of full-length nascent protein from 50Sof post translation ribosome, and in that process, its toe prints on the rRNA *in vivo* and in in vitro translation with *E*.*coli* S30 extract. We reported earlier that a number of chemically unfolded proteins like bovine carbonic anhydrase (BCA), lactate dehydrogenase (LDH), malate dehydrogenase (MDH), lysozyme, ovalbumin etc., when added to free 70Sin lieu of the full length nascent proteins, also interact with identical RNA regions of the 23S rRNA. Interestingly the rRNA nucleotides that slow down release of the C-terminus of full-length unfolded protein were found in close proximity to the B2a/B2b bridge. It indicated a potentially important chemical reaction conserved throughout the evolution. Here we set out to probe that conserved role of unfolded protein conformation in splitting the free or post-termination 70S. How both the RRF-EFG dependent and the plausible nascent protein–EFG dependent ribosome recycling pathways might be relevant in bacteria is discussed here.

## Introduction

In bacteria, termination of protein synthesis takes place when Class I release factor recognizes a stop codon on the mRNA. Then the nascent protein is cleaved off from the peptidyl tRNA, generating the post-termination complex consisting of 70S ribosome, the mRNA and the deacylated P-site tRNA. The release factors in *E*.*coli*, RF1/RF2 are released by RF3 to allow RRF to independently occupy ribosome in presence of EFG and IF3 [1–5]. They carry on “ribosome recycling”, the “fourth step” of protein synthesis, on the post-termination ribosome to initiate a fresh round of translation. RRF alone has been shown to dissociate the 70S partially in a cryo-EM study [6]. The GTPase activity of EFG plays crucial role along with RRF in dissociation, and IF3 sequesters the freed 30S subunit [7] to stabilize the dissociated state [4]. The gene encoding RRF (*frr*) is essential for bacteria [8]. Its deletion causes unscheduled translation termination [1]. However no proper eukaryotic homolog of it has been identified yet.

*In vitro* ribosome dissociation studies with RRF, EFG-GTP & IF3 [3–5] were performed on model post-termination complexes synthesizing oligo peptides of two to four amino acids long. Cleaving off those peptides by release factors or puromycin generated post-termination ribosome that act as the substrate for RRF, EFG-GTP and IF3. Above studies assumed identical molecular structure of post-termination ribosomes, whether synthesizing small oligo peptides or full-length proteins. Albeit a number of studies [9, 10] showed interaction of nascent protein with the wall of the peptide exit tunnel, leaving the possibility of slow release of nascent polypeptide from the ribosome. A full length nascent protein, tagged by C-terminal His, isolated from growing bacterial cell as well as from *in vitro* translation reaction was found associated predominantly with the 50S subunits, and a little with the 70S [11, 12]. Nucleotides of 23SrRNA interacting with that nascent protein are present in the close proximity of conserved inter-subunit bridge B2a/B2b joining the 50S and 30S [12]. The fact that RRF also interact at the same site [13, 14]may indicate that access of those nucleotides for RRF is only possible once nascent protein leaves the 70S; however isolation of predominant 50S population bound to full length nascent protein in fact lead us to question its role together with that of RRF in dissociating the 70S ribosome.

Here we revisit the ribosome-recycling step to check the contribution, if any, of the unfolded conformation of full-length nascent protein (which contains significant secondary structures formed co-translationally, but is not completely folded at the tertiary level) in splitting the post-termination ribosome. In our model system we compared splitting of 70S and (70S-tRNA) complex in the presence of full-length unfolded protein, RRF, EFG-GTP, IF3 along with known ribosome binding antibiotics. How far our *in vitro* data of light scattering and sucrose density centrifugation agree with the ribosome recycling studies done using *in vitro* translation system as well as *in vivo*, and how would that correspond to the known structure-function of ribosome, is discussed here.

## Materials & Methods

### Chemicals and strains

Test proteins–Carbonic Anhydrase from Bovine erythrocytes (BCA), Chicken egg white lysozyme, Ovalbumin from egg white, Malic Dehydrogenase (Mitochondrial) from Porcine Heart (MDH) and Lactate dehydrogenase from Pig muscle (LDH) were purchased from Sigma; guanidine hydrochloride was purchased from Sigma. 70S ribosome from *E*.*coli* MRE600 was prepared as described earlier [15–17]. Plasmids containing RRF, EFG and IF3 genes under T7 promoters [18] were kindly provided by Prof. Umesh Varshney, IISc, India. *E*.*coli* (pKR15) cells used for isolation and purification of tRNA^Glu^ were a kind gift from Dr. Jack Lapointe, University of Laval, Quebec, Canada. GTP, its non-hydrolyzable analogue GMPPNP, FITC (Fluorescein-5-isothiocyanate), Isopropyl-β-D-thiogalactopyranoside (IPTG), DEAE cellulose and the antibiotic fusidic acid were purchased from Sigma. BIOGEL P-60 and P-100 gel medium were purchased from BIORAD. The synthetic deca-peptide VGDANPALQK was a kind gift from Prof. D.K.Chattoraj, NIH, USA. *E*.*coli*LJ14 (temperature sensitive strain for RRF) was a kind gift from Dr. Santanu Dasgupta, Uppsala University, Sweden.

### Unfolding of test proteins

**Fig 1:**
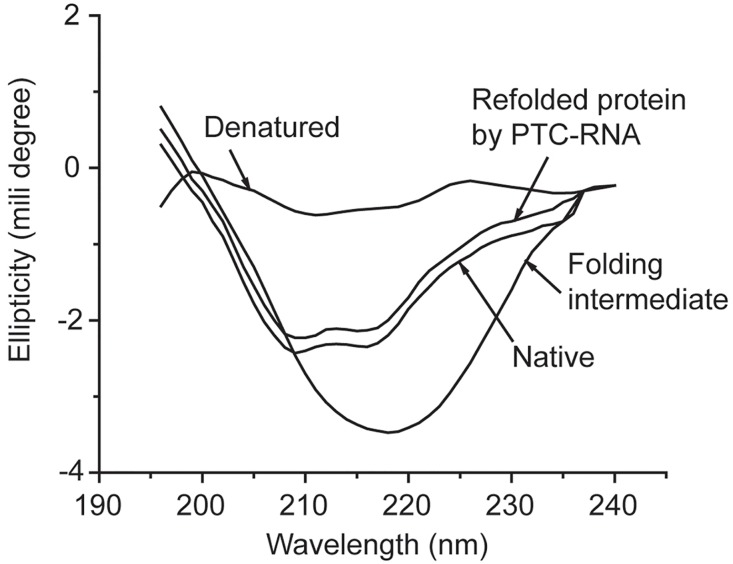
CD spectra of native bovine carbonic anhydrase (BCA), denatured BCA, folding intermediate of BCA and refolded BCA (by the PTC-RNA). Refolding was done following previous protocol [19], where guanidine denatured BCA was diluted 100 times in the buffer containing 50mM Tris (pH:7.5), 10mM Magnesium acetate and 100mM NaCl, at 25°C temperature, in presence of 200nM of PTC-RNA. To isolate the folding intermediate, denatured BCA was snap chilled after 2 hours of denaturation reaction and aliquoted out at requisite concentration of denatured protein into the above buffer immediately prior to CD measurement.

Unfolding of the test proteins BCA (Bovine carbonic anhydrase), Bacterial HspH, Lysozyme, Ovalbumin, MDH (Malate dehydrogenase) and LDH (Lactate dehydrogenase) were carried out as reported earlier [19–21]. In short, all native proteins were denatured using 6M guanidine hydrochloride for specified time as reported at 25°C temperature. Losses of secondary structures were monitored by CD spectrum (Fig 1).

### Purification of RRF, EFG, IF3

RRF, EFG and IF3 were purified using the protocol from Prof. Umesh Varshney, IISC, India [18]. In short, plasmids containing those genes were transformed into *E*.*coli* BL21 (DE3). Overnight cultures of these cells in LB medium were diluted and the fresh culture was induced with 0.5 mM IPTG. The overproduced proteins were purified using DEAE ion exchange and gel filtration (using proper BIOGEL medium, from BIORAD) column chromatography. All proteins were purified to apparent homogeneity as observed by single band for 5 μg protein in SDS-PAGE stained with silver. Purified proteins were stored in storage buffer (10mM Tris–HCl, pH 7.5; 50mM NaCl and 7mM MgCl_2_) containing 50% glycerol.

### Ribosomal subunits dissociation studies

Dissociation of 70S by the translation factors in presence and absence of unfolded protein was measured by sucrose density gradient centrifugation and by tracing the light scattering intensity following previous report [4, 20] at 20°C temperature. This lower temperature was chosen to slow down the process, so that the time course could be followed.

5%-20% Sucrose density gradient centrifugation was done at 48000 rpm for 45 minutes in Hitachi CS120GXL Ultracentrifuge. Optical Density of fractions at 260 nm were plotted to analyzed ribosome profiles.

**Fig 2:**
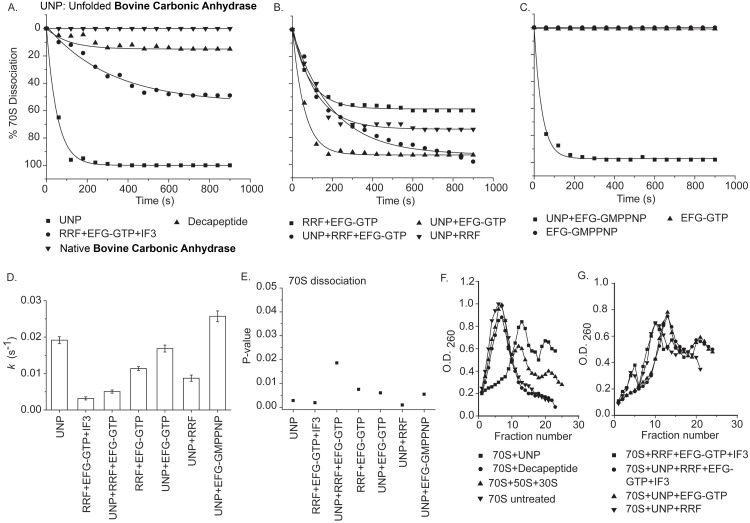
Dissociation of ribosomal subunits analyzed using fluorescence light scattering (at 20°C) and 5–20% sucrose density gradient centrifugation. **(A)**Percent (%) dissociations of 70S are plotted as a function of time by: unfolded bovine carbonic anhydrase (UNP) (■); RRF+EFG-GTP+IF3 (●); decapeptide VGDANPALQK (▲); Native bovine carbonic anhydrase (▼). **(B)**Percent (%) dissociations of 70S by: RRF+EFG-GTP+IF3 (■); UNP+RRF+EFG-GTP+IF3 (●); UNP+EFG-GTP (▲); UNP+RRF (▼) are plotted against time. **(C)**Percent (%) dissociations of 70S by: UNP+EFG-GMPPNP (■); EFG-GMPPNP (●); EFG-GTP (▲) are plotted against time. **(D)**Dissociation rate constants (*k;* s^-1^) are derived from the single exponential fits of the respective graphs and plotted as bar graphs against the corresponding combination of factors indicated in the figure. Error bars (s.d.) are propagated from three independent experiments for each combination of factors. **(E)** P-values for the dissociation rate constants (*k*) are calculated from three independent experiments for the respective combination of factors as indicated in Fig D and plotted here. Results showing statistical significance at *p*< 0.05. **(F)** Sucrose density gradient centrifugation showing dissociation of 70S by: unfolded protein (■); deca-peptide VGDANPALQK (●). The profile of 70S, 50S, 30S (▲) ran in a parallel sucrose gradient; and only untreated 70S (▼) ran in another gradient in parallel, are shown. **(G)** Sucrose density gradient centrifugation showing dissociation of 70S by: the combinations of RRF, EFG-GTP and IF3 (■);UNP, RRF, EFGGTP and IF3 (●); UNP and EFG-GTP (▲);UNP and RRF(▼).

**Fig 3:**
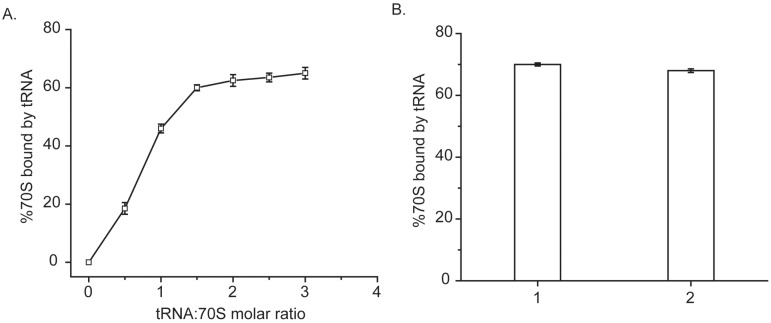
Filter binding of tRNA^Glu^ to 70S. **(A)** Percent (%) 70S (*E*.*coli* wild type) bound to the [α-^32^P] UTP labeled tRNA are plotted in Y-axis against tRNA: 70S molar ratio. **(B)** After binding 70S to [α-^32^P] UTP labeled tRNA at 25mM Mg^2+^, reaction mixture was diluted to7mM Mg^2+^ in the subunit dissociation buffer. Bar diagrams show percent (%) 70S bound by [α-^32^P] UTP labeled tRNA before (bar 1) and after (bar 2) dilution. Error bars (s.d.) are propagated from 3 independent experiments for each of the bars.

**Fig 4:**
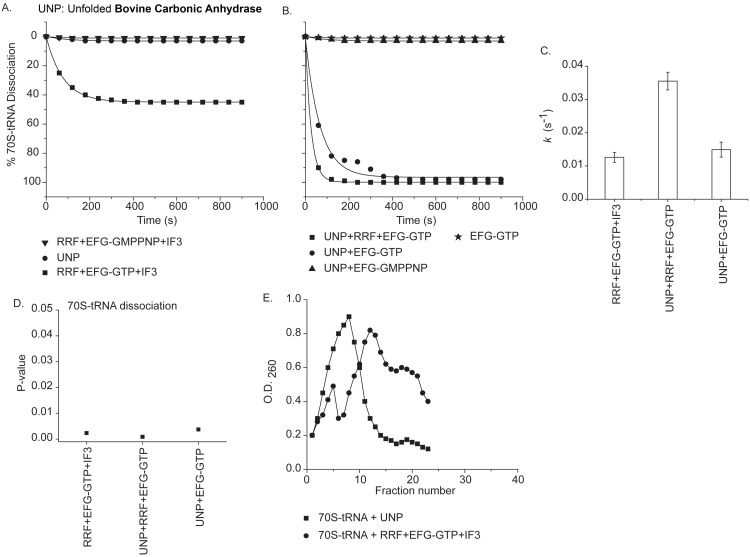
Dissociation of ribosomal subunits analyzed using fluorescence light scattering (at 20°C) and 5–20% sucrose density gradient centrifugation. **(A)** Percent (%) dissociations of (70S-tRNA) complex are plotted as a function of time by: unfolded bovine carbonic anhydrase (UNP) (●); RRF+EFG-GTP+IF3 (■) and RRF+EFG-GMPPPNP+IF3 (▼). **(B)**Percent (%) dissociations of (70S-tRNA) complex by: UNP+RRF+EFG-GTP (■); UNP+EFG-GTP (●); UNP+EFG-GMPPPNP (▲); and EFG-GTP (★). **(C)** Dissociation rate constants (*k;* s^-1^) are derived from the single exponential fits of the respective graphs and plotted as bar graphs against the corresponding combination of factors indicated in the figure. Error bars (s.d.) are propagated from three independent experiments for each combination of factors. **(D)** P-values for the dissociation rate constants (*k*) are calculated from three independent experiments for the respective combination of factors as indicated in Fig C and plotted here. Results showing statistical significance at *p*< 0.05. **(E)** Sucrose density gradient centrifugation showing dissociation of (70S-tRNA) complex by: unfolded bovine carbonic anhydrase (UNP) (■) and RRF+EFG-GTP+IF3 (●).

**Fig 5:**
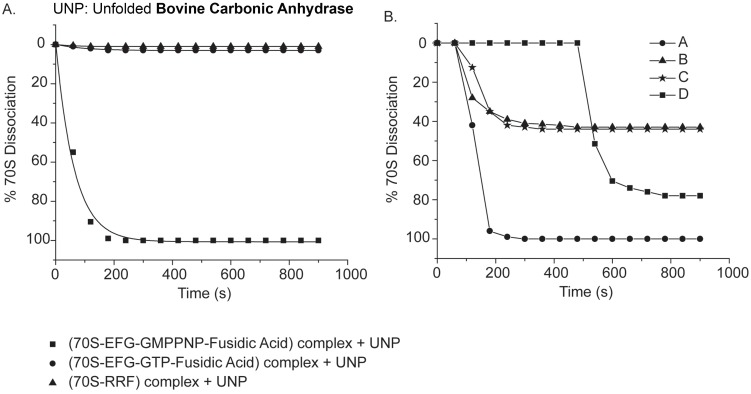
Dissociation of factors pre-bound to 70S, analyzed using fluorescence light scattering (at 20°C) and 5–20% sucrose density gradient centrifugation. **(A)** Percent (%) dissociations of 70S by unfolded bovine carbonic anhydrase (UNP) are plotted as a function of time from (70S-EFG-GMPPNP-fusidic acid) complex (■); (70S-EFG-GTP-fusidic acid) complex (●) and (70S-RRF) complex (▲). **(B)** Light scattering measurements after sequential addition of factor(s) to the 70S and 70S-translation factor complexes: [A] 70S pre-bound to EFG-GMPPNP (0 min) → addition of UNP (at 1 min.); [B] 70S pre-bound to EFG-GMPPNP (0 min) → addition of RRF at 1 min → addition of UNP at 8 minute; [C] Only 70S (0 min.) → addition of RRF at 1 min. → addition of UNP at 8 minute; [D] 70S pre-bound to EFG-GTP (0 min.) → addition of RRF at 1 min. → addition of UNP at 8 minute.

Light Scattering experiments were performed in the Hitachi F-3010 Fluorescence Spectrophotometer (excitation: 3 mm slit; emission: 3 mm slit; wavelength at 350 nm at 90° angle) using the following subunit dissociation buffer: 10 mMTris–HCl, pH 7.5; 80 mM NH_4_Cl; 7 mM MgCl_2_; 0.2 mM DTT. To the pre-incubated factor mix, 70S was added in the above buffer as specified in Figs 2–4 and recording of the scattering intensity started 3s after the mixing. In the final reaction volume 70S concentration was 0.1 μM. Amounts of the translation factors used were in five time molar excess of the 70S in all subunit dissociation experiments. For experiments where unfolded protein was added in combination with the translation factors, its concentration was also 5 times that of 70S used. Recording of the scattering intensity started within 5s of the mixing of ribosome and unfolded protein with the pre-incubated translation factor mix. For experiments where factor(s) pre-bound 70S ribosome(s) was used, 70S was previously allowed to bind with the factor(s) fusidic acid, RRF, GMPPNP and EFG-GTP as specified in Fig 5 following the previously reported protocols [13, 22, 23]. Similar procedure was followed to monitor the subunit dissociation kinetics.

### Binding of tRNA^Glu^ to the P and E site of 70S

tRNA^Glu^ was isolated and purified following published protocol [24]. In order to observe binding of tRNA to the 70S, [α-^32^P] UTP labeled tRNA was incubated with the 70S at 37°C for 30 min using the following buffer: 50 mM Tris-HCl (pH: 7.5), 100 mM NaCl, 20 mM / 25 mM MgCl_2_. As reported earlier, at 25 mM Mg^2+^ tRNA binds to both the P and E site of 70S and at 20 mM Mg^2+^ it binds to only P site of 70S [25]. We used cellulose nitrate filter paper with a pore size of 0.45 micron for filter binding assay to check tRNA binding to ribosome. Ribosomes are too large to pass through this filter paper. Free tRNA molecules would pass through and 70S bound tRNA molecules will retain on the filter paper. At twice the molar concentration of the ribosome, the tRNA more or less saturated the ribosome. Hence, in all our experiments, we added tRNA at three times molar excess of 70S to prepare 70S-tRNA^Glu^ complex. Following the preparation of this complex, we diluted it with a dilution buffer containing 160 mM of NH_4_Cl, so that after addition of pre-incubated factor mix into it, the subunit dissociation reaction took place in the dissociation buffer (10 mM Tris–HCl, pH 7.5; 80 mM NH_4_Cl; 7mM MgCl_2_; 0.2 mM DTT.).

### Binding studies on dansyl-lactate dehydrogenase (dansyl-LDH) with the intact 70S ribosome and the 50S subunit by fluorescence polarization measurements

LDH (Pig muscle, 1mg/ml) was reacted with 80-fold molar excess of dansyl chloride (dissolved in dry acetone), in buffer containing 10mM potassium phosphate pH 7.6 containing 20 mM KCl, at 0°C for 1 hour. The unreacted dye was removed by gel filtration on Sephadex G-50 in presence of 10 mM β-marcapto ethanol at 4°C. The LDH-conjugate (dansyl-LDH) was kept at 0°C for 10–12 hours and no appreciable change in the enzymatic activity was found. Molar ratio of dansyl to LDH was determined to be 1.3 assuming molar absorptivity of dansyl to be 4600 [26]. Steady state fluorescence was measured using a Hitachi F 3010 spectro-fluorimeter in a quartz cuvette of 10 mm path length. The temperature was kept constant at 20°C. Dansyl fluorescence was measured upon excitation at 330 nm when native dansyl-LDH showed an emission maximum at 505 nm. Polarization measurements were performed using Hitachi polarization accessory with excitation (EX: 330 nm) and emission (EM: 505 nm) slits of band pass of 5 nm and 10 nm respectively. Polarization values (P) were calculated from Chen & Bowman [27, 28]. For denatured dansyl-LDH, polarization measurements were done after 1 minute refolding in the buffer, 20 mMTris-HCl, pH 7.5, 10 mM Mg-acetate and 5 mM β-marcapto ethanol containing different concentrations of the 70S ribosome and its 50S subunit. The change in dansyl polarization with increasing concentrations of the 70S and 50S subunit were analyzed by a model independent method and the apparent dissociation constants of both 70S and 50S binding to dansyl-LDH both in native and denatured form (*K*_*d*_) were determined by nonlinear curve fitting analysis using the Eq (2) elaborated earlier [29]. It may be noted here that the measurements with 70S were done quickly after adding proteins to avoid significant dissociation of it. All experimental points for binding isotherm were fitted by least-square analysis using MS Origin software package (Origin Lab). The binding stoichiometry for both denatured and native LDH to the 70S and 50S particle was found to be ~1, estimated from the intercept of two straight lines of the nonlinear fitted plot of ΔP/ΔP_max_ against the input concentrations of the 70S and 50S subunit (not shown).

$$K_{d}=\left[C_{0}-\left(\frac{\Delta P}{\Delta P_{max}}\right).C_{0}\right].\frac{\left[C_{r}-\left(\frac{\Delta P}{\Delta P_{max}}\right).C_{0}\right]}{\left(\frac{\Delta P}{\Delta P_{max}}\right)}.C_{0}$$ (1)

$$C_{0.}{\left(\frac{\Delta P}{\Delta P_{max}}\right)}^{2}-\left(C_{0}+C_{r}+K_{d}\right).\left(\frac{\Delta P}{\Delta P_{max}}\right)+C_{r}=0$$ (2)

In Eqs (1) and (2), ΔP is the change in polarization for each point on the titration curve. ΔP_max_ denotes the same when LDH is completely bound to the 50S subunit, C_r_ being the concentration of the 50S subunit, and C_0_ is the initial concentration of LDH (0.04 μM). The double reciprocal plot was used for determination of ΔP_max_ and also the apparent binding constant (K_app_ = 1/*K*_*d*_) using Eq (1).

$$\frac{1}{\Delta P}=\frac{1}{\Delta P_{max}}+\frac{1}{\left[K_{app}\Delta P_{max}C_{r}\right]}$$ (3)

The linear double reciprocal plot of 1/ΔP against 1/C_r_ is extrapolated to the ordinate to obtain the value of ΔP_max_ from the intercept. The dissociation constants reported in this work are mean ± Standard Error of Mean (SEM) from four independent measurements (S1 Fig).

### *In vivo* protein folding assay

For in vivo protein folding assay [30], *E*.*coli* LJ14 [1, 8] temperature sensitive cells (transformed with a plasmid bearing *LacZ* gene) were grown at 28°C in Tris-Glycerol-Casamino acid (TGC) medium: 100 mM Tris-HCl (pH 7.5), 100 mM potassium chloride, 8.5 mM sodium chloride, 20 mM ammonium chloride, 1 mM calcium chloride, 1 mM magnesium sulphate, 0.3% casamino acid, 15 mM sodium pyruvate, 1 mM potassium phosphate (pH 7.5), 0.2% glycerol, 0.2 μg/liter ferrous sulphate and 10 μg/liter thiamine. In the log phase (OD_600_ = 0.2) cells, β-galactosidase was induced with IPTG. After 8 minutes of growth, cells were divided into two four equal portions. Three portions were allowed to grow at 30°C, one as control and the other two with the 30S specific antibiotic streptomycin (17 μM) and 50S specific antibiotic chloramphenicol (480 μM) respectively and the fourth part was grown at 42°C without antibiotic. At different time points aliquots were withdrawn from the growing cells and were lysed with toluene to measure their β-galactosidase activities. The 42°C sample was kept at that temperature for 10 minutes before aliquotes were taken, to ensure depletion ofthe active RRF [1, 8]. To 200 μl of the assay buffer [100 mM sodium phosphate (pH 7.5), 10 mM potassium chloride, 1 mM magnesium sulfate, 50 mM β-mercaptoethanol and 1 mg/ml ONPG], an equal volume of toluene extracts of cells grown under different conditions were added, and the mixture incubated for 30 seconds at 37°C. The reaction was stopped by adding 200 μlof 1M sodium carbonate and the enzyme activity was measured by recording OD_420_. β-galactosidase activities were normalized with respect to cell concentration before plotting as a function of time (S2 Fig). The difference between the beta-gal activities in cells to which 50S inhibitor antibiotic was added (which inhibits synthesis as well as nascent protein folding) and to which 30S inhibitor antibiotic (which inhibit synthesis but not nascent protein folding) was added, is the folding of the amount of beta-gal protein associated with the 50S at the instant of adding antibiotics (30). The increase in beta-gal activity after ten minutes at 42°C in the *ts* RRF cells should be due to RRF independent release of nascent folding protein from ribosome synthesized after temperature shift.

## Results

### Model system to study the role of full-length polypeptide in 70S ribosome dissociation

Toe-printing and MALDI-ToF MS/MS studies revealed that the 23SrRNA interacts with a nascent protein through the same nucleotides as it does *in vitro* (in presence of Mg^2+^) with the chemically unfolded form of the same protein when added “in trans” to 70S [12, 19]. A number of unfolded proteins, e.g., bovine carbonic anhydrase (BCA), lactate dehydrogenase (LDH), malate dehydrogenase (MDH), lysozyme, ovalbumin etc., have been shown to interact with the same nucleotides as well. This observation indicated towards a chemically conserved mechanism evolved from the RNA world [31] that led us to use chemically unfolded full-length protein as model polypeptide to study ribosomal subunits dissociation in presence of canonical ribosome recycling factors. Both the chemically unfolded protein and nascent full-length protein possess the conformation of unfolded polypeptide. Nascent protein attains considerable secondary structure co-translationally [9, 32], while in our experimental set up chemically unfolded protein assumes correct secondary structure when denaturant is diluted in presence of 70S. This secondary structure could be trapped as a ‘folding intermediate’ containing predominantly alpha-helix (Fig 1)–capable of interacting with 70S. CD spectra for different conformational states of Bovine Carbonic Anhydrase (BCA) shows disruption of secondary structures in presence of denaturant, presence of considerable secondary structures when folded by ribosomal RNA and its native state (Fig 1).

### Unfolded polypeptide dissociates free 70S ribosome

At 1 mM Mg^2+^, 70S dissociates completely into 50S and 30S subunits [4]. Fluorescence light scattering was used to follow the dissociation kinetics [4], which is highly temperature dependent [20]. At 37°C unfolded proteins split 70S within the mixing time to make it difficult to compare different dissociation kinetics using fluorescence light scattering [20]. So we selected our experimental temperature at 20°C. Dissociation of 70S in 1mM Mg^2+^ was taken as 100% and accordingly, the percent (%) dissociations in all the experiments were plotted as a function of time.

Unfolded BCA completely dissociated 70S at a very fast rate compared to slow and partial dissociation of 70S by RRF, EFG-GTP and IF3 in identical condition (Fig 2A and 2D–2G). Kinetic rate constants for 70S splitting were derived from single exponential fits of the splitting data and are shown as bar diagram in Fig 2D. Multiple repetitions showed statistical significance of the corresponding rate constants at *p*< 0.05 (Fig 2E). It may be kept in mind that since unfolded proteins, which start folding as soon as freed from denaturants, stopped flow kinetics could not be applied here. The rates etc. of dissociation were all measured at 20°C and are slower compared to other published literature [5, 33]. Unlike previous reports [3–5] a recent report [33] showed the optimal concentration of EFG for ribosome splitting to be ~5μM. While the concentration of all translation factors used here is 0.5μM that is 5 times in molar excess of the 70S used (0.1μM). To check possible physiological relevance of the above observation, unfolded protein was used in presence of RRF, EFG and IF3. RRF & EFG-GTP combined could split 70S [3–5] with a rate given in Fig 2B and 2D–2G. When unfolded protein was added to this combination (Fig 2B and 2D–2G), interestingly it did not dissociate the 70S at such a fast rate; even the rate appeared to be slower than that done by the combination of RRF & EFG-GTP (Fig 2B, 2D and 2E). Similarly, when unfolded protein was added with only RRF, rate of unfolded protein mediated dissociation of 70S slowed down, presence of EFG-GTP in that combination could not help to increase the rate (Fig 2B, 2D and 2E). While in absence of RRF from the above combination, unfolded protein along with EFG-GTP dissociate 70S at such a fast rate as it does alone (Fig 2A, 2B, 2D and 2E); replacing GTP by its non-hydrolyzable analogue i.e. EFG-GMPPNP did not affect that fast rate (Fig 2C, 2D and 2E). So, the unfolded protein mediated splitting of 70S was hindered by RRF and not by the EFG-GTP; indicating a competition between unfolded protein and RRF to dissociate 70S, perhaps one excluded the other in binding to 70S.

### Dissociation of P & E-site tRNA bound 70S

To mimic the condition at translation termination when the class I release factor(s) hydrolyze the peptidyl-tRNA, leaving deacylated tRNA at the P-site, we pre-bound 70S with deacylated tRNA at its P or both P and E sites [25]; confirmed by filter binding assay. The [α^32^P] UTP labeled tRNA (~75 nucleotides) passed through the cellulose nitrate filter paper with a pore size of 0.45 micron; but tRNA-ribosome complex became trapped on it (Fig 3A). The 70S-tRNA complex formed at high Mg^2+^ was stable when diluted to the subunit dissociation buffer (7mM Mg^2+^), confirmed by retention of P^32^ count upon filtration (Fig 3B). Unlike the case of vacant 70S, unfolded polypeptide alone failed to split the P & E site tRNA bound 70S (Fig 4A and 4E). The combination of RRF, EFG-GTP and IF3 was capable of splitting 70S-tRNA complex (Fig 4A, 4C and 4D), but replacing GTP with its non-hydrolysable analogue GMPPNP completely abrogated that (Fig 4A). This agreed with all previous reports on ribosome recycling [3–5]. Then we checked whether the factors that displace deacylated tRNA from the ribosomal P-site [3–5] could assist unfolded protein to dissociate tRNA bound ribosome. Unfolded protein in association with EFG-GTP and RRF dissociated the 70S from the tRNA complex at a comparable rate as it did to the 70S alone (Fig 4B–4D). Interestingly, the rate of splitting of the complex by unfolded protein and EFG-GTP in this occasion becomes faster in the presence of RRF (Fig 4B–4D). The main difference observed between RRF, EFG-GTP mediated splitting to that of unfolded protein, EFG-GTP mediated splitting of the complex is in the extent of splitting, which is ~100% in presence of unfolded protein in the combination (Fig 4A–4E). This can be attributed to the lower RRF and EFG concentrations used in our experiments; though unfolded protein even at this concentration can split the complex completely in presence of EFG-GTP (Fig 4A, 4B and 4E). In presence of EFG-GMPPNP, the unfolded protein failed to split the 70S-tRNA complex (Fig 4B), similarly as RRF is unable to dissociate 70S-tRNA with EFG-GMPPNP and IF3 (Fig 4A). Presumably an EFG-GTP mediated structural transition of 70S-tRNA complex leading to dissociation of tRNA [13, 14] is necessary for RRF or unfolded protein to split 70S.

### Dissociation of 70S pre-bound with factors

These studies were further extended by pre-binding one factor with the 70S and observing the effect of others in the dissociation reaction. Binding conditions of different factors with 70S were picked up from previous reports [13, 34, 22, 23].

**Fig 6:**
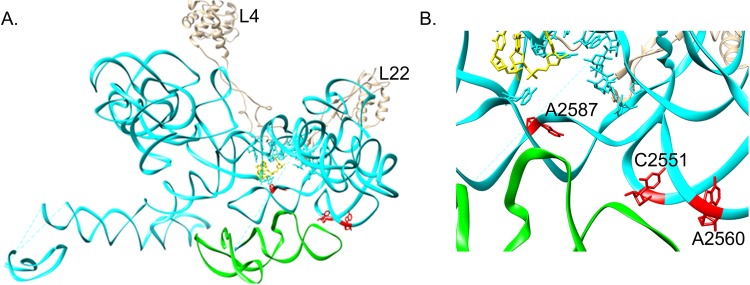
*E*. *coli* ribosomal RNA (rRNA) structure derived from PDB structure 3J7Z. **(A)** Domain V of rRNA is shown in cyan; two r-proteins L4 and L22 are marked that forms narrowest constriction at the peptide exit tunnel; CCA-end of the P-site tRNA is colored yellow; rRNA nucleotides that release unfolded protein slowly are marked red (based on our previous study [12]); rRNA helices that constitute important bridges B2a/B2b, B3 –that join 50S to 30S are marked green (RRF interacts with this region to dissociate 70S). **(B)** Close-up view showing rRNA nucleotides numbering that release unfolded protein slowly (red) are in close proximity to the rRNA helices constituting B2a/B2b, B3 bridges (green); CCA-end of tRNA is marked yellow.

Unfolded protein could not split 70S pre-bound to RRF (Fig 5A); close proximity of RRF and nascent protein binding sites on the ribosome (Fig 6) might explain this non-synergism. When we pre-bound EFG-GTP with fusidic acid, that locks EFG-GDP on 70S [35, 36], the unfolded protein was unable to dissociate 70S (Fig 5A). When GTP was replaced by GMPPNP, unfolded protein did split 70S-EFG-GMPPNP-fusidic acid complex (Fig 5A); presumably due to inability of fusidic acid to lock EFG in its GDP bound conformation on 70S.

As shown in Fig 5B **[A]**, in case of sequential addition of factors, unfolded protein added to the 70S-EFG-GMPPNP complex at 1 min dissociated the complex at the same rate as it dissociates the free 70S. When added only RRF to the 70S at 1 min, interestingly it itself dissociated 70S partially (Fig 5B **[C]**) and the addition of unfolded protein to this after an additional 7 min, produced no further subunit dissociation. Since RRF was present in stoichiometric amount, after dissociating a population of ribosome, probably it remained bound to the un-dissociated population of 70S and prevented the access of unfolded protein [6]. A similar result was observed when we replaced 70S by 70S-EFG-GMPPNP complex and then added RRF followed by the unfolded protein (Fig 5B **[B]**). However RRF could not split pre-formed 70S-EFG-GTP complex and addition of unfolded protein to this after 7 min resulted in ribosome dissociation at as fast a rate as it does to free 70S (Fig 5B **[D]**). These results go in favor of the previous reports where structural studies revealed that RRF, EFG share some overlapping binding sites on the ribosome and it is the EFG that pushes RRF during their concerted action for ribosomal subunit dissociation [13, 14]. Thus RRF and unfolded protein access their respective binding sites on ribosome through different routes. Although EFG-GTP plays no role when added along with the unfolded protein in splitting free 70S ribosome, in case of 70S-tRNA complex the presence of EFG-GTP was essential to remove the tRNA from 70S. So the splitting of factor bound 70S explained further that the unfolded protein mediated pathway of ribosome splitting is distinct from the RRF mediated one. How could the two parallel ribosome recycling pathways possibly be relevant in a bacterial cell, is discussed later.

### Comparing affinities of unfolded protein for 70S and 50S

We estimated the binding affinities of lactate dehydrogenase (dansyl-LDH) for both the 70S and 50S by fluorescence polarization (S1 Fig). Change in fluorescence polarization due to binding of native and unfolded LDH to the 50S at 20°C are shown in S1 Fig. Dissociation constants were found to be comparable for the same with chaperone proteins like GroEL [37]. Dissociation constants (*K*_*d*_) summarized in Table 1 show that the affinities of unfolded protein for both the 70S ribosome (20 ± 4 nM) and 50S (45 ± 5 nM) were comparable to Gro-EL and an order of magnitude higher than that of the native LDH (*K*_*d*_ = 289 ± 12 nM for 70S and *K*_*d*_ = 416 ± 15 nM for 50S). Binding was about 2-fold stronger with 70S compared to 50S. This in vitro observation with chemically unfolded proteins corroborated with the in vivo data where nascent unfolded (probably partially folded co-translationally) protein was traced to remain associated post-translationally with the 70S that synthesized it and not associated to a free 50S subunit in trans [12, 38]. We argue that this post-translational association of nascent protein to the 70S indeed results into dissociation of that 70S to its subunits.

**Table 1:** Affinity of ribosome and chaperones for unfolded proteins.

Proteins	Folding modulators	*K*_*d*_ *(nM)*
Denatured LDH	*E*.*coli* 70S ribosome	20 (this study)
Native LDH	*E*.*coli* 70S ribosome	289 (this study)
Denatured LDH	*E*.*coli* 50S ribosome	45 (this study)
Native LDH	*E*.*coli* 50S ribosome	416 (this study)
Denatured LDH	*E*.*coli* chaperonin GroEL	20 (see ref. [47])
Unfolded Proteins	*E*.*coli* DnaK	10–1000 (see ref. [48])
Nascent Protein Lep	*E*.*coli* Trigger Factor	311 (see ref. [49])

Dissociation constants (*K*_*d*_) for various unfolded and native proteins binding to ribosomes and chaperones are shown in the table. Affinity measurements in present study are done at 20°C temperature.

### Activity gain of unfolded protein—*in vivo* and *in vitro*

If the unfolded/full length nascent protein mediated recycling evolved distinctly from the RRF mediated one, then the activity gain of nascent full-length protein (and not aborted translation product), yet to dissociate from the post-termination ribosome, should be independent of RRF in vivo. To check this, an experiment was performed which we designed several years ago [30] to demonstrate the role of large ribosomal subunit in folding/activity gain of nascent protein. While bacterial growth was severely compromised the activity gain of beta-galactosidase was impressive in the temperature sensitive strain of RRF (*LJ14*) [1, 8] growing at non-permissive temperature (42°C) (compare S2A and S2B Fig). The activity gain of the population of nascent protein synthesized just prior to the temperature shift from 30°C to 42°C has been shown in S2B Fig; where samples were collected after 10 minutes at 42°C to ensure depletion of RRF in those cells [1, 8]. In parallel, the population of nascent protein synthesized just prior to the addition of 30S specific antibiotic streptomycin at 30°C showed considerable increase in beta-gal activity while the sample treated with 50S specific antibiotic chloramphenicol at 30°C showed no gain of beta-gal activity under identical condition (S2B Fig) [30]. As we reported years ago, although the addition of commonly used 30S and 50S specific antibiotics stop protein synthesis in the cell within a minute, the ribosomal 50S in such cells can fold the still un-dissociated proteins in presence of 30S specific antibiotics, but not in presence of 50S specific antibiotics. So, the difference in beta-gal activity in 50S and 30S specific antibiotic treated cell is a measure of in vivo protein folding by the 50S subunit. The increase in beta gal activity after 10 minutes incubation at 42°C where ts-RRF could no longer act in ribosome splitting, could be explained by saying that the full-length nascent protein could dissociate from the ribosome without assistance from the RRF.

## Discussion

At the end of an authentic translation termination in bacteria, class 1 release factor (RF1/RF2) recognizes stop codon on the mRNA and triggers peptidyl tRNA hydrolysis to free the nascent full-length protein as well as the last P-site tRNA. Then the deacylated tRNA is dissociated from 70S in presence of EFG-GTP by RRF [3–5] or unfolded protein [20]. EFG-GTP to EFG-GDP transformation is associated with a structural transition of 70S—a prerequisite for tRNA dissociation. In a recent cryo-EM report [14] it has been shown that RRF promotes the rotated state [39]of ribosome and destabilizes the H69 and H44, thereby EFG-GTP facilitates RRF reorientation and actively splits the B2a bridge connecting 50S and 30S (Fig 6). Thus a common aspect of EFG function at the molecular level has been reflected in both elongation and ribosome recycling phases of translation. While in another study [12] comparing interaction of nascent unfolded protein and chemically unfolded protein with the rRNA also revealed differential affinity of rRNA nucleotides to the different regions of the unfolded protein(s). Those rRNA nucleotides identified to release slowly the C-terminal region of the protein are located in close proximity to the B2a bridge (Fig 6). Hence splitting of 70S by unfolded protein can be conceived as the regulation of rRNA nucleotides that slowly release the unfolded polypeptides at the end of translation and EFG-GTP functions identically as it does during translation elongation to promote disruption of B2a bridge. Present study indicating the ability of both the RRF and the unfolded protein to dissociate tRNA from the ribosome by interacting along with EFG, also point towards that mechanism. The observation that the structure of C-terminal end of nascent peptide is important in translation termination [40] is also relevant in this regard. Because of the independent role of unfolded protein in tRNA dissociation, it can remain bound post-translationally to the ribosome even after its dissociation into subunits. That is why we get a large population of nascent full length protein bound to the 50S both *in vivo* and in the translation system with the S30 extract [11, 12]. Moreover, our present study of ribosome recycling with the chemically unfolded protein also point towards a chemically conserved interaction between rRNA and unfolded conformation of protein that has evolved from the RNA world [31].

Question arises—why should there be two parallel ribosome recycling pathways, especially since RRF is an indispensable protein in bacteria [8]and RRF mutants are lethal. Almost all the *in vitro* studies done to establish the RRF, EFG mediated recycling pathway, have used the model post-termination complex where small oligo-peptides and not the full-length proteins [3–5] were synthesized. Protein synthesis in bacterial cell terminates not only at the authentic termination sites, but also profusely by premature termination after synthesizing oligo and short polypeptides [41]. Different cellular components have evolved in prokaryotes and eukaryotes to rescue those stalled ribosomes [41, 42]. Similarly RRF, EFG mediated ribosome recycling pathway would be indispensable for recycling so many prematurely stalled ribosomes. This possibility has already been checked and reported by a number of independent groups [41–46]. It has been suggested that, *in vivo*, RRF and EFG can recycle the ribosomes that are stalled in elongation due to delay in recruitment of aminoacyl tRNAs for rare codons or lack of a stop codon in truncated mRNAs [43]. Thereby the presence of another recycling pathway in bacteria as an intrinsic ribosomal activity has been suggested [44]. As for the nascent folding protein, splitting of 70S ribosome and sequestrating the 50S can be viewed as a counterpart of sequestration of 30S by IF3.

Instead of using the model post-termination complex we used 70S bound P and E site tRNA complex in the present study. By comparing our results with the experiments on model post-termination complexes [3–5] we find that the translation termination factors RRF, EFG-GTP and IF3 react identically in both the cases. The rate of splitting of ribosome in our experiment was slower due to lower experimental temperature. As mentioned before, this could not be avoided since we could not use the stopped flow technique here. In order to observe the characteristic kinetics of chemically unfolded protein mediated 70S splitting, one has to start monitoring the kinetics within a few seconds of the addition of unfolded protein to 70S. Delaying addition of folding protein to the rRNA by even half a minute could spoil the experiment because the folding protein would go through “unassisted folding” by this time. This is why stopped flow technique could not be applied to the present study as such time limits could not be followed there for mixing unfolded protein into the reaction buffer.

We checked secondary structural elements in the unfolded protein that is the substrate of ribosome or rRNA and compared that with the product (folding protein) as well as that of native protein. Fig 1 shows that the substrate of ribosome for folding contains considerably higher secondary structure, while the product is as usual like native protein. Nascent protein is also believed to contain considerable secondary structure (formed co-translationally) while inside the ribosome. The rate of translation of a message is not uniform throughout its length. It has been demonstrated that pauses during translation allow segments of protein chains to form local secondary structure [32]. The pause sites could be at rare codons, which are decoded by low-abundant tRNAs. When we checked the amino acids of different proteins that interact with the nucleotides of PTC RNA [12, 19](Peptidyl Transferase Center–rRNA), not all were coded by the rare codons. However, the relative codon abundances were generally low in the regions in which those amino acids were present (S3 Fig). Thus, the secondary structure formation of nascent protein chains arising from translation pauses [32] might also be responsible for guiding the secondary structure dependent binding of amino acids to the rRNA nucleotides.

In conclusion, in the present article we have demonstrated how translation termination and ribosome recycling might be linked with the folding of the translation product. The role of IF3 and unfolded proteins in sequestering the 30S and 50S subunits respectively also ensures their short-lived independence to gear up for the next round of translation.

## Supporting Information

S1 Fig Binding of *E*. *coli* 50S subunits with dansyl labeled (A) native LDH and (B) denatured LDH (0.04 μM). Plots show increase in the dansyl polarization in presence of increasing concentrations of 50S subunits at 20°C. Insets show the respective double reciprocal plot of the changes in dansyl polarization for both native and denatured LDH against the 50S subunit concentrations.
(TIF)

S2 Fig Beta Gal assay in *rrf*-TS mutant. (A) *E*.*coli* cell density was measured for LJ14 (*rrf* TS) strain growing in TGC medium at 30°C and 42°C. (B) The cells, grown at 30°C, was divided in four parts at 0 min and induced with IPTG. Three of them were subsequently grown at 30°C, without and with antibiotics streptomycin and chloramphenicol respectively and the fourth one was grown at 42°C. The time when antibiotics were added and also ten minutes past temperature shift to 42°C, is shown in the figure with an arrow mark. Subsequently, β galactosidase activities were measured in all of them.
(TIF)

S3 Fig Codon frequency: per thousand plot. Using codon usage database, Codon freq.: per thousand are plotted for four proteins–BCA (A), Ovalbumin (B), Lysozyme (C) and HspH (D) as a function of corresponding codon numbers; positions of amino acids that interact with the PTC-RNA for folding are marked by asterisks.
(TIF)
