# Supplementary figures and images for: A Possible Role of the Full-Length Nascent Protein in Post-Translational Ribosome Recycling
Source: PLoS One. 2017 Jan 18;12(1):e0170333. doi: 10.1371/journal.pone.0170333 (PMC5242463; doi:10.1371/journal.pone.0170333)

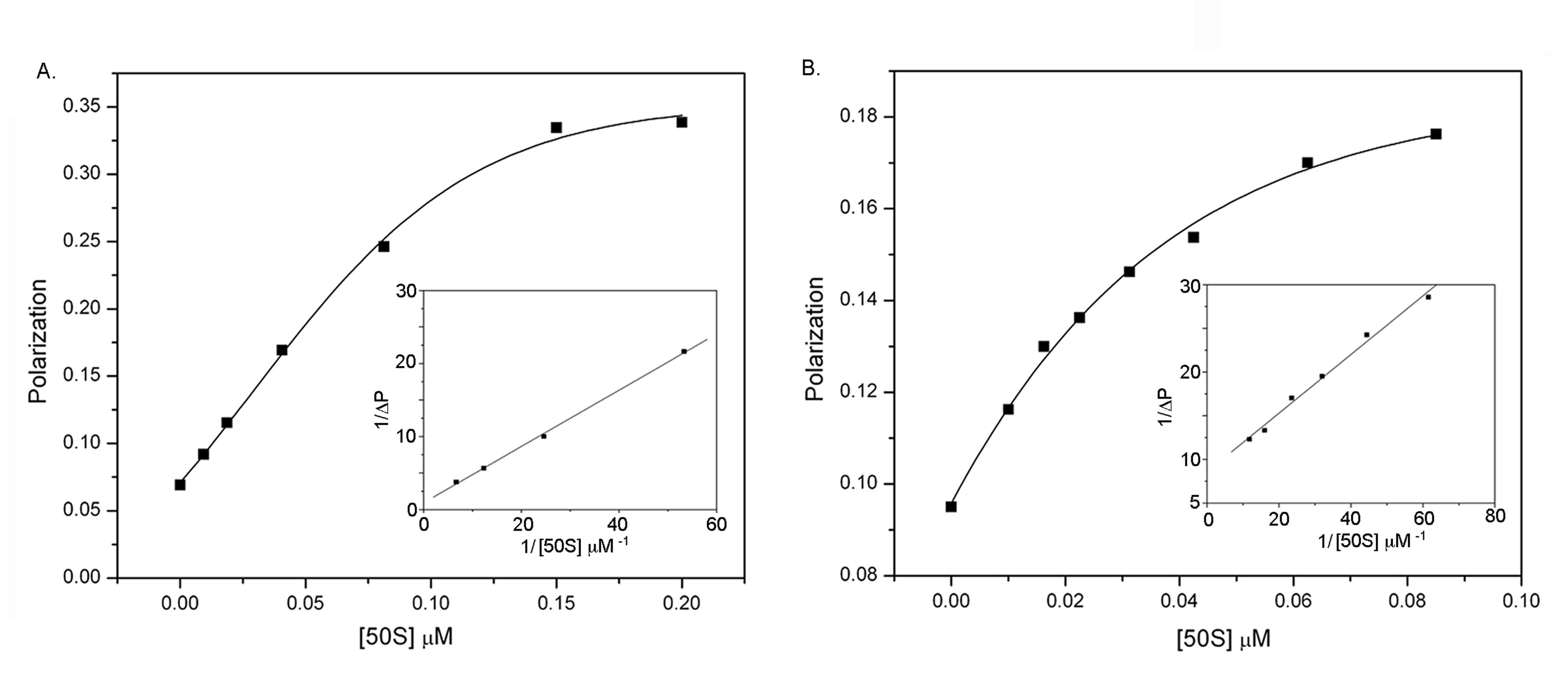

Supplement: S1 Fig — Plots show increase in the dansyl polarization in presence of increasing concentrations of 50S subunits at 20°C. Insets show the respective double reciprocal plot of the changes in dansyl polarization for both native and denatured LDH against the 50S subunit concentrations. (TIF) [file pone.0170333.s001.tif]

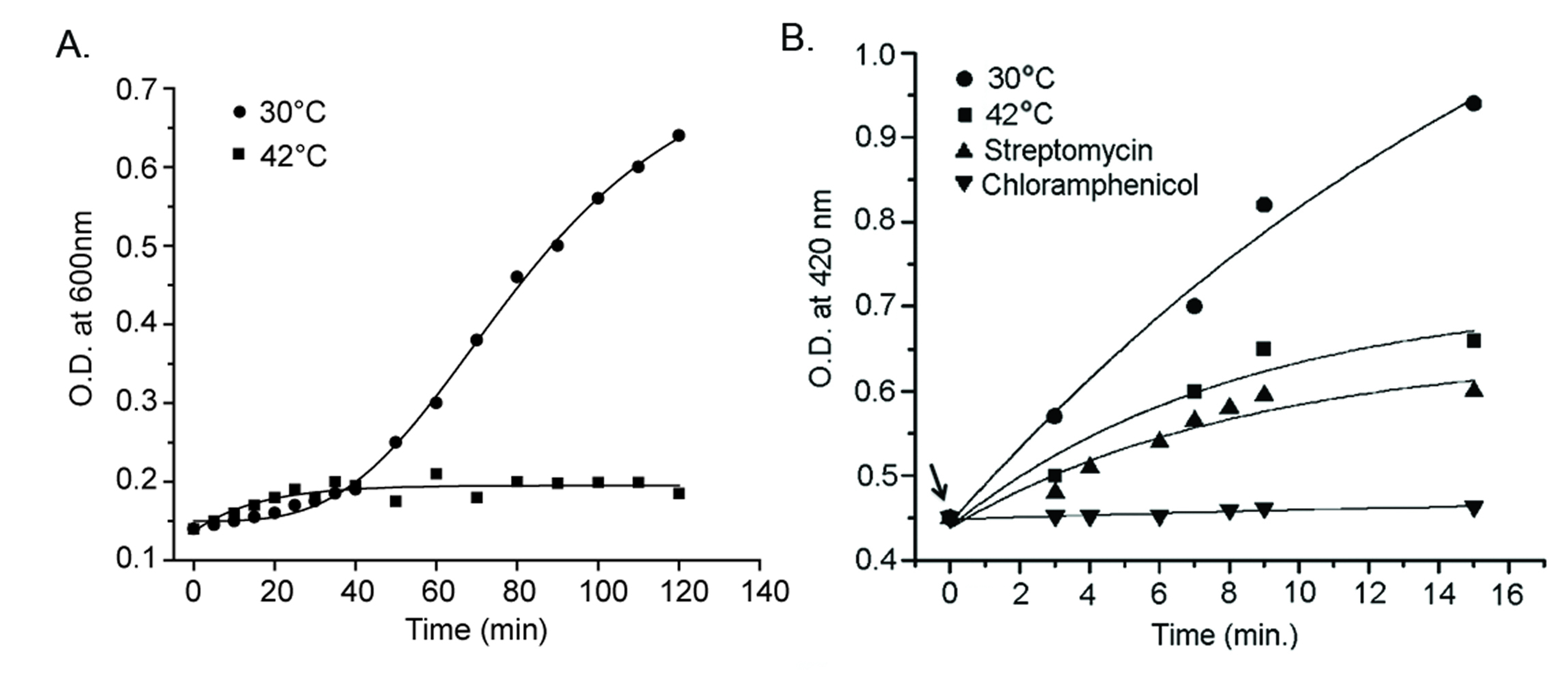

Supplement: S2 Fig — (A) E.coli cell density was measured for LJ14 (rrf TS) strain growing in TGC medium at 30°C and 42°C. (B) The cells, grown at 30°C, was divided in four parts at 0 min and induced with IPTG. Three of them were subsequently grown at 30°C, without and with antibiotics streptomycin and chloramphenicol respectively and the fourth one was grown at 42°C. The time when antibiotics were added and also ten minutes past temperature shift to 42°C, is shown in the figure with an arrow mark. Subsequently, β galactosidase activities were measured in all of them. (TIF) [file pone.0170333.s002.tif]

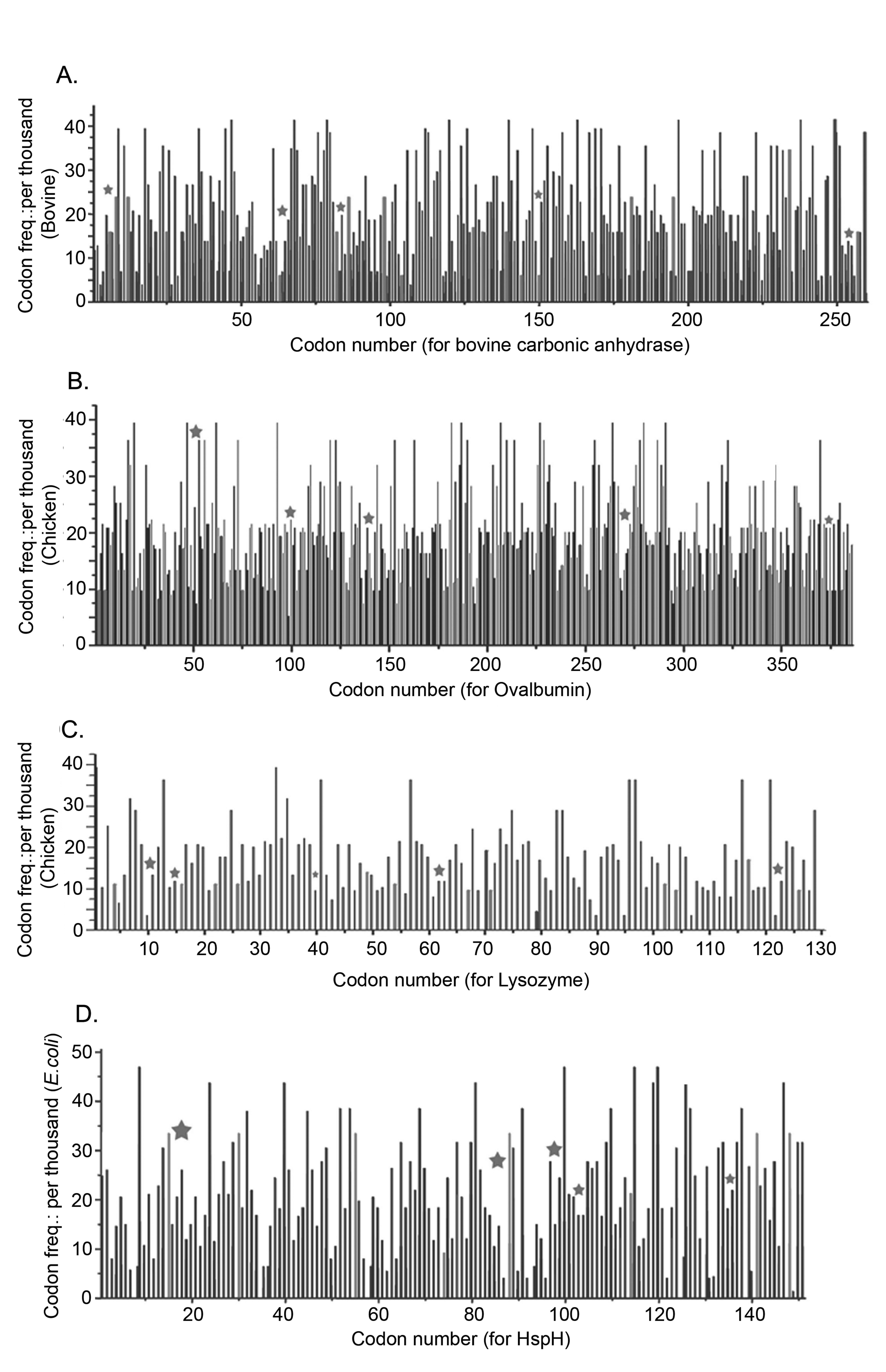

Supplement: S3 Fig — Using codon usage database, Codon freq.: per thousand are plotted for four proteins–BCA (A), Ovalbumin (B), Lysozyme (C) and HspH (D) as a function of corresponding codon numbers; positions of amino acids that interact with the PTC-RNA for folding are marked by asterisks. (TIF) [file pone.0170333.s003.tif]
